# The Evolution of Empathy and Women’s Precarious Leadership Appointments

**DOI:** 10.3389/fpsyg.2015.01751

**Published:** 2015-11-12

**Authors:** John G. Vongas, Raghid Al Hajj

**Affiliations:** Department of Management, John Molson School of Business, Concordia University, Montreal, QC, Canada

**Keywords:** glass cliff, empathy, leadership, evolutionary psychology, cultural evolution

## Abstract

Glass cliffs describe situations in which women are promoted to executive roles in declining organizations. To explain them, some authors suggest that people tend to “think crisis-think female.” However, the root cause of this association remains elusive. Using several subfields of evolutionary theory, we argue that biology and culture have shaped the perception of women as being more empathic than men and, consequently, as capable of quelling certain crises. Some crises are more intense than others and, whereas some brew within organizations, others originate from the external environment. We therefore propose that women will be selected to lead whenever a crisis is minimal to moderate and stems primarily from within the organization. Men, on the other hand, will be chosen as leaders whenever the crisis threatens the very existence of the firm and its source is an external threat. Leadership is a highly stressful experience, and even more so when leaders must scale glass cliffs. It is imperative that we understand what gives rise to them not only because they place women and potentially other minorities in positions where the likelihood of failure is high, but also because they help propagate stereotypes that undermine their true leadership ability.

## Women and Leadership: From Glass Ceilings to Glass Cliffs

Today, more than ever before, women who pierce the glass ceiling are joining the ranks of executive leadership once considered the sole province of men. Despite this progress, few of them hold top positions in government, business, law, and medicine (Anderson and Court, 2012). A global study on the percentage of board seats occupied by women in the largest companies found that women held 19.2% of seats in the US (S&P 500) and 20.8% in Canada (S&P/TSX 60) (Catalyst, 2015). In Europe and Asia-Pacific, respectfully, their participation ranged from 7% in Portugal (PSI-20) to 35.5% in Norway (OBX Index), and from 3.1% in Japan (TOPIX Core 30) to 19.2% in Australia (S&P/ASX 200). Demand for gender equality in boardrooms is surging, and efforts requesting diversity through lobbies and quotas have peaked (Catalyst, 2014). For example, in Canada, a country known for labor-market gender equity, the Ontario Securities Commission recommended that companies listed on the Toronto Stock Exchange disclose how they recruit and select women executives (Canadian Press, 2014). Women’s managerial involvement has not been without its critics, however, some of whom have condemned women leaders for ruining firm performance:

“So much for smashing the glass ceiling and using their unique skills to enhance the performance of Britain’s biggest companies. The march of women into the country’s boardrooms is not always triumphant—at least in terms of share price performance. Analysis of FTSE 100 shares shows that companies that decline to embrace political correctness by installing women on the board perform better than those that actively promote sexual equality at the very top.” (Judge, 2003, p. 21)

At best, the above claim published in the British press could be seen as a mismatch between society’s moral obligation to give women a fair shot at leadership and women’s actual performance as leaders. At worst, it could be interpreted as a mismatch between one’s gender and one’s leadership ability. Stated plainly, women’s “unique skills” as organizational leaders are simply ineffective. Given its controversy, this claim led to the burgeoning of studies exploring the relationship between organizational performance and leader gender (for a comprehensive review, see Bruckmüller et al., 2014). Findings from this research unearthed evidence that women breaking through the glass ceiling must overcome an added hurdle in their quest to attain executive roles—that of scaling a *glass cliff*.

Ryan and Haslam (2005) coined the term glass cliff to describe situations in which women, more so than men, are promoted to executive positions in companies with declining performances. After scrutinizing Judge’s (2003) original FTSE 100 data, and comparing 15 companies with female board member appointments to 16 with male appointments, they found that while men tended to be selected during stable stock market conditions, women were chosen when market conditions were depressed in the 5 months preceding the appointments. The authors argued that women were more likely than men to assume executive positions in firms that were already associated with poor performance thereby showing preliminary evidence that board of directors seemed to be biased in favor of female leaders during crises. This “think crisis-think female” heuristic meant that these women were perched on a fragile glass cliff because their roles entailed a greater risk of failure. As such, the authors considered the attack to be unjust because women inherited firms with less stability compared to men. To discover more evidence, Haslam and Ryan (2008) then carried out the first experimental studies on glass cliffs by asking graduate management students (Study 1; *N* = 95), high school students (Study 2; *N* = 85), and business leaders (Study 3; *N* = 83) to select an individual from a list of candidates to lead either a thriving or a failing hypothetical firm. Consistent with their original prediction, participants across studies were more likely to appoint a female leader when the business was declining rather than improving. Moreover, Haslam and Ryan (2008) provided the first empirical evidence that people from different life stages and occupations harbor similar mental associations linking women leaders to crisis situations. In recent years, other scholars have found support for glass cliffs. For example, Mulcahy and Linehan (2014) compared 138 firms that experienced a financial loss to those that reported a profit during a 3-year period from 2004 to 2006. Specifically, they explored whether changes in gender diversity had taken place in the firms’ board composition following the release of financial results. As expected, firms having reported a financial loss witnessed an increase in the number of women hired on their boards compared to those that had reported a profit.

We argue that, similar to the way in which glass ceilings have represented gender inequality in promotion opportunities, glass cliffs can now be seen as representing gender inequality in assignment opportunities. Notwithstanding the discriminatory practices that women have had to endure, including their nomination for precarious leadership positions, our society’s current moral stance on the need for unbiased decision making in the workplace is not only making such practices more noticeable, but it is also asking us to confront them with greater urgency. Therefore, this mismatch between a social environment which was once supportive of an exclusive male hegemony and a current one that condemns such an unfair advantage constitutes one possibility that may explain why glass cliffs are now coming to the fore as heated ethical dilemmas. But what explains the decision-making bias to select women leaders in times of crisis? Our primary purpose is to offer an original explanation for women’s glass cliff appointments by first asking: Are humans predisposed to perceive women—and by extension women leaders—as being more empathic than men and, consequently, as nurturing figures that are sought for support during a crisis? Our secondary goal is to show that not all organizational crises are isomorphic and thus distinguish not only between the types of crises that occur, but also the intensity or severity with which they unfold. If women are said to possess qualities that enable them to deal with crises, then investigating contextual variation in glass cliffs in these two respects seems warranted. Finally, we broaden our framework to encompass two other Darwinian perspectives through which glass cliffs could be analyzed, namely behavioral ecology and cultural evolution.

In the sections that follow, we first define empathy and provide an exposé of evolutionary psychology meant to explain its biological etiology. Second, we present evidence of women’s empathic advantage over men and explain the implications of this individual difference for organizational leadership in crisis situations. Third, we explore factors that describe under which conditions women leaders are likely to be selected for glass cliff appointments. Fourth, we usher in other evolutionary approaches to behavior in an effort to show how these can enrich the discussion surrounding the onset of glass cliffs. Lastly, we reiterate the importance of evolutionary theorizing and people’s need to be aware of their subconscious biases before concluding with some ideas for future exploration and implications for managerial practice.

## The Evolutionary Basis of Empathy

Derived from the Greek *empatheia* (where *en* = “in” and *pathos* = “feeling”), empathy is defined as “the capacity to be affected by and share the emotional state of another, assess the reasons for the other’s state, and identify with the other, adopting his or her perspective” (de Waal, 2008, p. 281). Scholars first conceptualized empathy as a cognitive process (e.g., perspective-taking) before describing it in affective terms (e.g., distress), and gradually began approaching it as a multifaceted construct. The evolution of empathy has been compared with a Russian *matryoshka* doll where the inner core, believed to have developed first, is nested within a series of outer layers (de Waal, 2008). Emotional contagion, the most primitive core of empathy, involves the emotional synchrony between people. It is then followed by sympathetic concern, which is the concern one has about another’s state and any subsequent attempts made to ameliorate that state (i.e., consolation). Finally, perspective taking, or the capacity to understand another’s situation and needs as separate from one’s own, is considered to be the most outer layer of empathic development and the most recent one to have evolved.

What explains how and why empathy evolved in humans? Ever since Aristotle presented his “Four Causes,” the question of causality has been central to scientific inquiry. Inspired by Aristotelian logic, ethologist Nikolaas Tinbergen devised a system to better comprehend behavior, one that rested on four basic questions about causality known commonly as the Four Why’s (Tinbergen, 1963). These questions fall into two categories, one dealing with proximal explanations of behavior and the other addressing ultimate ones. The logical starting point when using evolutionary principles begins by observing behaviors to understand how each behavior is structured, and why it was selected for. Whereas proximate causation relates to how a behavior operates, ultimate causation refers to why a behavior exists in the first place. More specifically, proximate explanations deal with the structural machinery underlying the behavior (mechanism or causation) and with the behavior’s developmental change over time (ontogeny). Ultimate or evolutionary explanations, on the other hand, are concerned with the benefits the performer receives of having engaged in the behavior (adaptation or function) and the description of the behavior’s history in a particular species (phylogeny).

Researchers in evolutionary psychology have been divided on the basis of which category of Four Why’s to pursue when studying human behavior. Whereas some have focused primarily on (mostly cognitive) proximate mechanisms (e.g., Tooby and Cosmides, 1992), others endorse a holistic view that behavior is best understood by considering both proximate mechanisms and ultimate function (Dunbar and Barrett, 2007). Adherents of the former approach treat the mind not as a blank slate or tabula rasa but instead as an adaptive toolbox that evolved under ancestral environments (Pinker, 2002). Its intellectual spirit drew in part from the human sociobiology movement of the late 1960s and mid-1970s which explained that differences and similarities in behavioral patterns were related to fitness costs (Tooby and Cosmides, 2005). Like sociobiologists, evolutionary psychologists of this school contend that natural selection worked on the effects of behavior on individuals who interacted repeatedly over time (Trivers, 2002). During their course, however, selection pressures designed a multitude of specialized neural circuits or modules. Housed in the brain, these domain-specific modules act like information processing units whose operations are integrated to produce behaviors that help humans solve countless adaptive problems each affecting human survival and reproduction (Cosmides and Tooby, 1992). Therefore, whereas the level of explanation in sociobiology is behavior, the one in evolutionary psychology is the psychological mechanism (Smith, 2000).

Recent work by de Waal (2008) suggests that empathy may have evolved as a proximate mechanism for altruism. Individuals across cultures engage in cooperative behaviors, sometimes risking bodily harm or incurring other costs, such as time and financial resources (Wyman and Tomasello, 2007). Throughout history, helping within collective groups had been a function of the degree of genetic relatedness between helpers and recipients, with participants preferring to help their closest biological kin (Hamilton, 1964). For this reason, empathy is believed to have originated from parental care and is as phylogenetically ancient as humans themselves (Eibl-Eibesfeldt, 1972). Infants worldwide communicate their affective states through smiling and crying, and these signals act as vital cues for caregivers to respond (Bowlby, 1958). Therefore, parents who possessed an intuitive understanding of the feelings, emotions, and intentions of their infants were more likely to transmit their “empathy genes” to future generations, while those who were indifferent or unsuccessful at soliciting their needs risked losing them (Vongas, 2009). Indeed, the evidence on parental responses to infant distress points to a conclusion that empathy triggers parental sensitivity and subsequent caregiving behaviors (Murray, 1979).

Natural selection may also account for the evolution of empathy among individuals who share no familial ties. According to the theory of reciprocal altruism (Trivers, 1971), selection acted on the recurring interactions between non-kin members who forged long-term exchange relationships to facilitate each individual’s fitness. Thus, psychological mechanisms for offering help and assistance to non-relatives were able to evolve so long as support was mutually reciprocated in the future. Should the survival and reproductive benefits that one party received be larger than the costs the other sustained in providing help, then those who engaged in this type of reciprocation out-reproduced those who did not, causing this kind of helping design to proliferate throughout a population. According to Trivers (1971), among the parameters that are required for reciprocally altruistic behaviors to be selected for, a given species must depend on one another and interact repeatedly over time to assist in a myriad of duties, such as caring for the offspring of non-kin and enlisting each other’s help in combat. Natural selection therefore seems to have favored altruism among familiar individuals and previous cooperators—both kin and non-kin—and was likely biased against previous defectors (de Waal, 2008). Such altruism in response to another’s distress would not have developed in the absence of empathy (Plutchik, 1987). It remains to be seen, however, how evolution can explain sex differences in empathy and how we evolved to perceive and come to expect women to be more empathic than men and, allegedly, better suited to handle crises.

### Sex Differences in Empathy

Inasmuch as evolution through natural selection shaped the mind in relation to survival, evolutionary pressures also exercised their influence on the mind through sexual selection (Darwin, 1871/1890). According to this process, men and women ought to exhibit differences in domains where each sex has been faced with distinct adaptive problems. Reproduction is one such domain where the reality of one sex is different from that of the other; across cultural and temporal contexts, compared to men, women have experienced more hardship in childbirth and in ensuring the survival and safety of their children (Buss, 2005). A subtheory of sexual selection, parental investment theory (Trivers, 1972), maintains that the sex that invests the most in the survival and subsequent raising of offspring should be more discriminating in mate choice, whereas the sex that invests less should compete more aggressively for mating opportunities with the higher-investing one. In other words, behavioral differences between women and men can be explained in part by the roles that each sex faced with respect to resources needed in parental care. Whereas men can choose to invest minimally in the future of their offspring (i.e., a simple act of intercourse), women must bear greater costs extending over a lengthier period of time (i.e., pregnancy sickness, gestation, morbidity, and mortality during childbirth, lactation). In light of their disproportionate costs in raising children and incurring greater costs with the selection of a poorly suited mate, women have evolved a capacity to be generally more sensible in their mating choices compared with men (Buss and Schmitt, 1993). Therefore, one could argue that women share a Darwinian heritage in being adept at imagining oneself in another’s situation and understanding another’s feelings, desires, and intentions. Because women typically invest more parental resources than men, having empathy would have been a key asset in this regard. By comparison, women deficient in empathy were more likely to experience interactions with partners and kin who failed to provide them with assistance needed in child rearing. To say that empathy was favored exclusively in women, however, is misleading. Empathy was selected for not only through parent-infant interactions involving mothers as primary caretakers (Preston and de Waal, 2002), but also through associations with other group members to facilitate forgiveness among exchange partners and to ensure future cooperation (Axelrod, 1984). Men have benefitted and continue to benefit from having empathy as parents and non-parents. The ability to understand others and experience their thoughts and feelings is tantamount in developing genuine social relations irrespective of sex or civil status (Batson, 1990). However, the mother-infant relationship appears to play a special role in fostering empathy as “infants are emotionally affected by the state of their mothers and mothers are emotionally affected by the state of their offspring” (Preston and de Waal, 2002, p. 7; also noted by Darwin, 1872/1998; Plutchik, 1987).

There now exists a panoply of studies from numerous disciplines on sex differences in empathy (Pinker, 2008). For example, over 40 years ago, Mehrabian and Epstein (1972) found that women were more empathic than men (*d* = 0.98), and suggested that separate statistics be used for each group. More than two decades later in a meta-analysis on sex differences in personality (*N* > 105,000), Feingold (1994) reported that women scored higher than men on tender-mindedness (*d* = 1.07), a construct known to overlap with empathy. Sex differences in empathy have also been observed indirectly in research on the Big Five model of personality. For example, in a study of 26 cultures (*N* > 23,000), Costa et al. (2001) found that women were more agreeable than men (*d* from 0.05 to 0.55). Big Five measures of agreeableness, as Nettle (2007) noted later, were equivalent to measures of empathy using the Empathy Quotient (EQ; Baron-Cohen and Wheelwright, 2004) with women scoring higher than men on both traits (*d* = 0.60 on agreeableness and *d* = 0.63 on EQ). These values are consistent with those of Lippa (2010) (*d* = 0.56, *N* > 250,000) although larger than the ones reported by Schmitt et al. (2008) (*N* > 17,500; mean *d* = 0.15). Finally, even when using a shortened version of the EQ scale and risking to compromise reliability, Andrew et al. (2008) found that women scored higher than men in empathy (*d* = 0.83).

Other studies not relying on traditional self-report questionnaires have also pointed to a female empathy advantage. For example, Connellan et al. (2000) presented newborns with a human face and with a mechanical object and found that, whilst male neonates showed a stronger interest in the object, females showed greater interest in the face. Other studies have revealed that women’s empathic superiority reflects the sexes’ different exposure to testosterone while in the womb (Baron-Cohen and Wheelwright, 2004). Longitudinal studies have shown that fetuses exposed to higher testosterone levels in utero make less eye contact as infants in their first year, resort to a smaller vocabulary in their second year, and socialize less with their fellow kindergarten classmates in their fourth year (Lutchmaya and Baron-Cohen, 2002; Knickmeyer et al., 2005). In another longitudinal study, Udry (2000) found that women subjected to more testosterone during embryonic development exhibited masculinized behaviors as adults despite their parents’ feminine-oriented socialization efforts. These findings complement others showing that females significantly outperform males in a host of behavioral and cognitive measures of empathy, such as sharing and turn-taking (Charlesworth and Dzur, 1987), responding to others’ distress (Davis, 1983), showing sensitivity to facial expressions (Hall, 1978), inferring what people might be thinking or intending (Baron-Cohen et al., 2001), and accurately recalling information about another person (Hall and Schmid Mast, 2008). In light of the theoretical reasoning and empirical evidence for the claim that women possess an empathy advantage, let us consider how this advantage translates to the realm of leadership.

## Women, Empathy, and Transformational Leadership

Research on leader empathy *per se* is beginning to gain momentum (Scott et al., 2010). In organizational behavior, this is surprising given that empathy has been characterized as “the *sine qua non* of all social effectiveness in working life” (Goleman et al., 2002, p. 63). One domain where women have outperformed men and in which their empathic advantage might be important is transformational leadership. Transformational leaders exert their influence by elevating their followers’ needs and objectives, and by affording them with the confidence needed to perform beyond their goals. They exhibit charisma, provide intellectual stimulation, motivate in inspirational ways, and nurse their followers with individualized consideration. Support for the claim that female leaders are more transformational than their male counterparts has been offered in conceptual (Bass, 1985), empirical (Vinkenburg et al., 2011), and meta-analytic studies (Eagly et al., 2003). According to Eagly et al. (2003), the primary sex difference in transformational leadership resides in women’s greater use of individualized consideration which involves supportive behaviors like encouragement and coaching. Such a consideration that helps develop followers’ confidence by attending to their needs would have been more difficult to enact in the absence of empathy.

Transformational leadership is thought to be particularly important in dynamic and unstable environments that rely on the need for change (Waldman et al., 2001). Given its plausible connection with empathy, one would expect a surge in research interest on gender differences in empathy as it relates to leading chaotic organizations described in the glass cliff. Over the last 25 years, some scholars have been proponents of a “female leadership advantage” and have argued that women are more likely than men to possess the values and abilities needed to manage modern organizations (Helgesen, 1990; Book, 2000). Accordingly, the changing nature of organizations demands that leaders espouse values such as nurturance, sharing, compassion, consensus building, and inclusiveness all of which are generally associated with female characteristics (Coughlin et al., 2005). Although we have argued that women’s empathic superiority relative to men stemmed in part from the differential selection pressures they faced, we are not advocating that women make superior leaders because of a relative advantage in this singular trait. Our goal was to present a framework and empirical findings that explain both why and how women express empathy more than men, on average, and that it is ultimately our perception of these women as being more empathic that contributes to glass cliff appointments. We now move to clarify the relationship between perceived female empathy and glass cliffs.

## Think Crisis, Think Female

In justifying why glass cliffs occur, Ryan and her colleagues provided five causes (Ryan et al., 2007). First, these are the result of either *hostile* or *benevolent sexism*. In the former, people desire to see women fail and attempt to actively disadvantage them or transform them into scapegoats (Ellemers et al., 2004), whereas in the latter they believe that they are doing women a favor by offering them challenges (Glick and Fiske, 2001). Second, glass cliffs might be due to in-group favoritism where male executives reserve attractive positions for their in-group members (Powell and Butterfield, 2002). Third, given women’s scarce prospects in upper managerial strata, high-risk leadership positions are among the few opportunities in which they can excel. Fourth, some companies wish to signal change by enacting gender equality (Wright et al., 1995). Finally, women are perceived as simply having the necessary socioemotional traits to handle crises. Each of these causes is a form of prejudice and, in times of distress, the authors suggest that people spontaneously engage in “think crisis-think female” or “think crisis-think *not* male” (see also Ryan et al., 2011).

In a recent comprehensive review of the literature on glass cliffs, Bruckmüller et al. (2014) concluded that these phenomena are indeed determined by multiple causes. To lessen their occurrence, they urged above all that top management cease to associate stereotypically feminine qualities with female leaders and to contrast these with masculine-like ones. This suggests that a principal driver of glass cliffs is the conviction that women are preferred as leaders during crises *because* they are possibly better equipped to deal with them than men. Although research agrees that this association exists, its nature has not been investigated directly. Women are said to possess crisis-relevant traits, abilities, skills, and behaviors (Mano-Negrin and Sheaffer, 2004; Bruckmüller and Branscombe, 2010; Ryan et al., 2011; Gartzia et al., 2012), yet researchers use these terms loosely thereby creating some obfuscation. The same confusion extends to how this advantage has been described. For instance, some have described women leaders as “being understanding,” “intuitive,” and “tactful” (Ryan et al., 2011, p. 477), “aware of the feelings of others” and “creative” (Haslam and Ryan, 2008, p. 542). Others have characterized them as “accentuating partnership” and “adopting empathic relations with subordinates” (Mano-Negrin and Sheaffer, 2004, p. 109). To explain why these gender-based leader perceptions occur, most concur that perceptions are guided “by broad societal expectations” (Rink et al., 2012, p. 1312) without asking what led to their formation. We believe that many of the adjectives used to describe women leaders fall under the broader purview of empathy. For example, highly empathic individuals have long been regarded as tactful and imaginative or creative (Hogan, 1969). Empathic accuracy is one’s ability to accurately infer another’s feelings and it is the aspect of empathy that corresponds to intuition (Greenson, 1977; Vongas and Al Hajj, 2015). Lastly, being understanding is central to the empathic experience because it relates to perspective taking, itself a key subdimension of empathy (Davis, 1980). Therefore, for these reasons, we believe that empathy is germane to understanding why women may be preferentially selected as leaders in times of crisis.

## Seeking Empathy in Crisis

Nearly 60 years ago, Bowlby (1958) postulated that children develop affective, cognitive, and behavioral schemas to ensure their proximity to primary caregivers or “attachment figures.” In seeking refuge between the empathic female or the comparatively less empathic male, those who chose the former would have had access to more resources and better care and thus a better chance to survive. This implicit female-empathy association would have been seared in the human subconscious and reinforced through observation and practice of seeking solace among primary caretakers (i.e., mostly mothers). This association would have also been strengthened by a social experience of being reared by networks of female caregivers assisting each other (Hrdy, 2000). Whereas women typically assign more importance to emotions than men in interpersonal relations, both sexes prefer to receive emotional support from women (Eagly, 2009). This affinity to gravitate toward women can be witnessed in neonates who favor the soothing voices of their mothers and of women in general to those of men (DeCasper and Fifer, 1980). Such evidence coming from individuals who have yet to be socialized portray the human mind as having a predilection for turning toward women for succor. In other words, it appears that women themselves not only evolved to possess design features that heightened their empathy relative to men, but humans in general have also evolved to perceive women as possessing empathic superiority. Hence, we believe that these two distinct but related processes are crucial in explaining why people seem to veer toward selecting women leaders in times of crisis and in explaining under what conditions such a preference may be the strongest.

An organizational crisis is any major threat to the survival of a system where response time is limited, the situation is ill-structured, and resources are inadequate (Mishra, 1996). Ryan and Haslam (2007) defined crisis as “any form of dramatic reduction in financial well-being that has an adverse bearing on the state of an organization” (p. 553). These definitions frame crises as classic engineering problems that require identifying and fixing operations that result in poor use of resources (Mitroff and Pearson, 1993). Recently, some scholars have insisted that these approaches direct attention away from how crises personally affect organizational members and disturb their social connections and attachments (Kahn et al., 2013). As such, they redefine a crisis by focusing on how relational systems are damaged and persist after the firm recovers, and advised that repairing crises “often requires repairing relational damage” (Kahn et al., 2013, p. 378). An effective strategy would require post-crisis leaders to convene followers so they could empathize with one another as they toil through troubling events. This, however, does not mean that board members will choose a less qualified leader to steer a company through a crisis because of the candidate’s sex. The pool of candidates for top positions is small as is the variability of candidates’ credentials. Also, given that companies are ill-prepared to deal with CEO succession in the wake of an emergency (Zajac, 1990), the ancestral prototype of the “leader-healer” in calamitous times may now become salient and favor women. This evolved proclivity toward women in times of crisis coupled with a low base rate of women considered for leadership positions will make their recruitment into precarious positions more visible and thereby open the glass cliff phenomenon to scrutiny. Not all crises are identical, however (Van Vugt and Spisak, 2008). We have proposed that humans evolved to seek women in need but if crises differ, and if the sexes have had different success rates in managing each of these types, then it would be reasonable to question whether men and women differ with respect to which crises they are perceived to be better suited to handle as leaders.

## Crisis Anatomy and Leader Preference

Any crisis seen as a direct threat to an organization’s existence could either come from the external environment as in the case of interorganizational conflicts (e.g., hostile takeover *between* companies) or the internal environment as in the case of intraorganizational turbulence (e.g., mistrust between unions and management *within* a company; Probst and Raisch, 2005). Given these crisis types, a question that bears to mind is which crises are more susceptible to women’s glass cliff appointments. One possibility is to try and understand how the process of natural selection helped create sex differences in the emergence of leadership during conflicts within and between groups. In one study, Van Vugt and Spisak (2008) subjected teams to engage in a public-goods game under conditions of either intragroup conflict or intergroup competition. They posited that intergroup competition would support the selection of a male leader, whereas intragroup conflict would favor that of a female leader. Their rationale was that each sex evolved unique adaptations to deal with problems in its respective role and that these adaptations would, in turn, elicit specific sex-biased leader prototypes in followers. Given that humans traditionally existed in collective groups with a rigid division of labor, the behavioral strategies that men and women employed benefited each sex differentially. Whereas men hunted and fought in wars, women foraged and invested greater resources for the birth and the upbringing of their children.

This reasoning led Van Vugt and Spisak (2008) to develop the male warrior hypothesis which holds that men evolved psychological mechanisms to facilitate coalition formation against rival outgroup members (see also McDonald et al., 2012). By comparison, it remained vital for women to invest resources in sustaining social networks to support themselves and their children (Taylor et al., 2000). Thus, women are thought to have a greater interest than men in maintaining their group’s integrity and this may explain their motivation to keep intragroup peace (Van Vugt et al., 2008). Conversely, men coalesce to dominate other groups because resources accrued from an intergroup victory elevate their status (Chagnon, 1988). In hunter-gatherer societies like the Ache of Paraguay and the Hadza of Tanzania, food acquisition extends beyond its functional significance and serves as a conspicuous signal to prospective mates (Hrdy, 2000). This drive for dominance is proffered to explain why men might be more willing and able than women to assume leadership roles during intergroup conflicts, and why they might be preferentially selected in crises of this sort (Van Vugt et al., 2007). Although humans today live differently than hunter-gatherers, competition remains a focal activity that can affect the fate of any social structure. By the same token, contemporary organizations resemble tribes of the past; both are social arenas where individuals compete with one another for the survival and welfare of their ingroup members. If one chooses to accept this notion, then one may expect the sexes to be elected in leader roles according to the type of crisis experienced.

Recent studies have looked more closely at the nature of organizational crisis and its effect on leader selection. In the first, Ryan et al. (2011) demonstrated that women are chosen to lead when the crisis requires managing people, bearing the brunt of failure, or enduring until the crisis subsides. Men, on the other hand, are chosen when the organization requires a spokesperson or when the goal is to reverse downward trends. Building on these findings, Rink et al. (2012) showed that participants evaluate risky leadership positions as a function of available resources. During a crisis, women appease people’s concerns, ride out the downturn, and may even act as sacrificial lambs and, as such, appear to be more interested in the communal aspects of leadership by focusing on social resources. Comparatively, men tend to be more involved in activities that improve company performance. Thus, they may favor financial resources over social ones when leading in a crisis. Studies have found evidence linking assertive traits that are associated with male leaders and communal traits associated with female leaders to think manager-think male and think crisis-think female heuristics, respectively (Sczesny, 2003; Atwater et al., 2004). In line with this logic and consistent with the finding that women prefer to manage people during a crisis than to concentrate on performance (Rink et al., 2012), we propose that women will be favored as leaders in an intraorganizational crisis while men will be selected as leaders in an interorganizational one, given equal qualifications on other attributes such as experience, competence, and reputation. We also believe that people’s ingrained perceptions of—and both personal and vicarious experiences with—empathic females and less empathic males play a key role in these leader choices. This prediction is congruent with the evolutionary-based female peacekeeper hypothesis which explains that individuals having feminine markings are more likely to emerge as leaders when efforts are needed to restore peace within a group (Spisak et al., 2012). Interorganizational crises can at times develop into intraorganizational ones, such as in the case of a price war between competing firms that leads to restructuring and layoffs. In such cases, we believe that a firm’s board of directors will determine who is best to lead the company according to the type of crisis they expect represents the most imminent danger.

Another important attribute that characterizes a crisis and that can impact women’s glass cliffs is crisis intensity or severity. Crisis intensity remains to be explored in glass cliff research and, as such, an assumption exists that women will be chosen to lead a crisis-laden firm irrespective of the crisis’s magnitude. Such a prospect requires a conceptual leap because of neglected context (Johns, 2006). To address crisis intensity, we used the term “glass cliff” to locate and peruse 64 studies published between 2005 and 2015. In the majority of experiments, crisis level was either unacknowledged or presented as minimal to moderate. For example, crisis was qualified variously as a “steadily decreasing performance in the past 5 years” (Ryan et al., 2011, p. 473), a “steady drop in financial performance,” (Haslam and Ryan, 2008, p. 533) or a “steady drop in appeal” (Haslam and Ryan, 2008, p. 536) with only one study describing the crisis as a “tremendous downward trend” (Bruckmüller and Branscombe, 2010, p. 436). Simply put, glass cliff researchers have not considered a threat so ominous that would risk jeopardizing the organization’s existence. In hostile environments, the roles that followers expect their leaders to assume are predictable, and include being less supportive and consultative and more assertive, directive, and decisive all of which suggest a stereotypically masculine autocrat (Mulder and Stemerding, 1963; Mulder et al., 1971).

Our predictions that female leaders will be selected over male leaders during intraorganizational crises, and male leaders over female ones during interorganizational crises, should hold whenever crisis intensity is unlikely to threaten firm survival. Several findings offer insight as to why empathy, a trait we have touted as being relevant for leaders during intraorganizational crises, will be less appealing when crisis intensity is extreme. First, while leader empathy is sought by followers who require comfort, understanding, and emotional support until the crisis begins to abate (Rink et al., 2012), the same trait is not likely to inspire those experiencing a calamity. Second, when the firm’s concern is focused on turning performance around from the brink of economic collapse, research has shown that stereotypically masculine traits in leaders (e.g., dominance) seem to be desirable over feminine traits (e.g., warmth; Ryan et al., 2011, study 3). In essence, what we are saying is that the relationship between an organizational crisis and women’s glass cliff appointments will be moderated by the type and intensity of the crisis, such that the relationship will be the strongest during intraorganizational crises of low-to-moderate intensity.

To recapitulate, a potential cause underpinning glass cliff appointments lies in the human tendency to seek the more empathic sex in distressful situations. Given that women were historically precluded from ascending to leadership positions in industrialized societies (Eagly and Carli, 2007), the idea that they confronted glass cliffs remained dormant until now. It is only when women became contenders for CEO positions that recruiters’ perceptual biases became triggered and produced what we now see as glass cliffs. A possibility remains that across temporal and sociocultural contexts, individuals sought comfort in primary caregivers most of whom were women and who possessed empathic qualities needed to manage distress. Why would there not be a similar mechanism operating in an organizational context, one that would call for choosing a female leader? It remains questionable whether this would be the case when a company is performing adequately. In this context, followers might shift their attention away from their leader’s communal traits and focus instead on agentic ones (e.g., dominance Eagly, 1987, 2009).

We have resorted to using evolutionary psychology as a theoretical framework to generate predictions about glass cliffs. Although some scholars praise its impact and popularity, the same scholars have criticized it for portraying humans as passive recipients of selection rather than active shapers of their cultural environments (Laland and Brown, 2011; Brown and Richerson, 2014). According to these critics, viewing evolution as a process through which the mind was influenced by environmental pressures says little about humans’ effort and ability to construct their niches as they see fit. Human behavioral ecology and cultural evolution are other Darwinian subfields each one treating culture in its unique way and adding to our understanding of glass cliffs. Although researchers typically focus on the distinctions between these subfields, some have argued that complementarity between them exists and should be pursued (Alcock, 2001). We discuss each of these subfields next to see how they could inform our rationale.

## The Multiple Lenses of Evolution

### Human Behavioral Ecology

Unlike evolutionary psychologists who argue that natural selection acts on the regulatory machinery (i.e., the psychological mechanism) that underpins behavior (Symons, 1987), human behavioral ecologists focus instead on how people’s behaviors are influenced by their environments, and how the adoption of alternative behaviors produces cultural differences (Borgerhoff Mulder, 1991). Their aim is to account for variation in behavior by questioning whether models of optimality and fitness maximization offer satisfactory explanations for differences seen across individuals (Laland and Brown, 2011). Also, whereas evolutionary psychology relies on participant self-reports in experimental settings, behavioral ecology observes people’s actual behaviors in field settings (Dunbar and Barrett, 2007). Human behavioral ecology’s two principal tenets are that humans exhibit behavioral flexibility that allows them to adapt across different environments, and that adaptive tradeoffs or compromises limit the extent to which they can pursue a strategy given available resources (Hawkes, 1996; Low, 2015). As such, selection would have favored the ability to take on strategies that maximized benefits and/or minimized costs in a given milieu, strategies that took the form “in situation A, do *x*, whereas in situation B, switch to *y*” (Smith, 2000). The precise manner in which environmental cues brings about a change in behavior may depend on innate dispositions or socially transmitted culture, but understanding these causal mechanisms is not a prerequisite to studying the fitness outcomes of particular strategies (Laland and Brown, 2011).

Behavioral ecologists could help to elucidate what gives rise to glass cliff appointments by observing the conditions under which women are chosen to lead whenever groups experience a crisis. By comparing the leader selection practices of various societies and their impact on subsequent group performance, they can determine whether it is optimal to choose women leaders when a crisis is intraorganizational (e.g., mistrust among ingroup members) as opposed to interorganizational (e.g., threat of attack by outgroup members). One possibility might be to compare the recruiting practices of modern industrialized societies with those of pre-industrialized ones that bestow both sexes with commensurate power and authority. Electing to study some societies in which women are considered as capable as men to assume leadership might be a prudent first step over exploring others that have a stricter division of labor. According to Low (2015), tribal and band societies that are characterized by women who wield significant influence include the Creek of the southern US, the Bemba of northeastern Zimbabwe, and the Ashanti of south Ghana. In each of these, men and women appear to have equal decision-making influence (see also Whyte, 1978, 1979). In crisis situations affecting either of these societies, researchers could model the costs and benefits for women being assigned to leadership roles. They could frame the study in terms of decision rules or conditional strategies such as the following: “If the crisis calls for mending interpersonal strife within the group and as long as it is not too intense, choose the female candidate; otherwise, if the crisis involves settling a dispute between neighboring groups, select the male candidate.” Testing hypotheses would then require using mathematical models to predict the optimal behavioral pattern in a given circumstance, and revising them to include other variables or tradeoffs to achieve better fit (Laland and Brown, 2011). As with all behavioral ecology models, to investigate glass cliffs, one would need to specify a *goal* (e.g., quell internal conflict or achieve intergroup victory), a *currency* that gages the costs and benefits of implementing the decision (e.g., difference in outcomes from choosing either leader), a set of *constraints* that characterize the context (e.g., crisis intensity), and a *decision* (e.g., leader selection, female or male; see Winterhalder and Smith, 2000). Behavioral ecologists would then be able to test Tinbergen’s hypotheses related to a behavioral pattern’s survival value or function by providing insight to the question, “what advantage(s) did choosing a woman leader in a particular crisis type and intensity provide the group’s descendants in the struggle to survive and reproduce?”

Some may argue that the sexes need not have equal power and authority for women to be preferentially chosen to lead during a crisis. Potential glass cliff processes merit investigation as long as women can be trusted to take on any leadership position in spite of its frequency, its degree of formality, or even its accompanying influence. This would also broaden the scope of settings in which one could directly observe and interview community members (for a comprehensive list of over 180 existing societies featuring remnants of earlier modes of living, see Betzig, 1986; also Low, 2015). Another possibility is to compare industrialized societies in the extent to which they promote women’s leadership during crisis situations. Research on work-related values has revealed that modern cultures differ in the differentiation of gender roles or what has become known as the masculinity-femininity cultural dimension of work values (Hofstede et al., 2010). While masculine societies support male dominance and economic performance (e.g., Japan, Mexico), feminine cultures accept gender-role fluidity and emphasize quality of life (e.g., Scandinavian countries; see also Hofstede, 1984). This cultural difference may help to explain the preponderance of women on Norwegian corporate boards (almost 36%) over those on Japanese boards (less than 4%; Catalyst, 2015). A recent meta-analysis examined the extent to which stereotypes of leaders are culturally masculine, and found that masculine construals of leadership are decreasing over time partly due to the increasing number of women leaders and, consequently, the propagation of a more androgynous concept of leadership (Koenig et al., 2011). Participation of women in national parliaments is on the rise globally (Inter-Parliamentary Union, 2015) and, while this may reduce bias toward current and potential female leaders, it might also exacerbate their likelihood of being given failing mandates.

### Cultural Evolution

The idea that culture consists of variants that compete with one another similar to the ways in which alleles or genotypes compete began with the work of human geneticists on cultural inheritance (Cavalli-Sforza and Feldman, 1973). Like behavioral ecologists, cultural evolutionists build models based on theoretical traditions of evolutionary biology but instead tailor them to the unique processes of culture. Since some cultural traits are more likely to spread than others, they can explain and predict patterns of change and diversity. Culture is defined as knowledge, beliefs, values, and attitudes capable of affecting individuals’ behavior acquired from others through teaching, imitation, and other forms of social transmission (Boyd and Richerson, 2005). Once culture is framed as “packages” of learned information, the processes by which variants of these packages change in frequency in a given population can be studied. Cultural variants differ from genes in some respects (Richerson and Boyd, 2005). First, they can be transmitted not only vertically from parents to offspring, but also obliquely from older to younger generations, and horizontally from one person to another (e.g., from siblings, friends, and peers). Second, unlike genes, some cultural variants are adopted by people while others are rejected, a process known as biased cultural transmission or cultural selection (Durham, 1991). Whereas biased transmission depends on the inner workings of the minds of cultural learners or imitators, natural selection depends on the extent to which different genes can survive and reproduce with little regard to human preferences. Cultural evolution is nevertheless a Darwinian process by which particular socially learned benefits, or pieces of knowledge, increase or decrease in frequency owing to being adopted by individuals at different rates (Cavalli-Sforza and Feldman, 1981). Evidence that cultural evolution is a biological process comes from studies showing that cultural information *varies* from person to person, *competes* for survival against other ideas, and is *inherited* by subsequent generations (Laland and Brown, 2011).

Most models in the social sciences postulate that gender differences in behavior emerge as a result of learning (Udry, 2000). Among the theories receiving the most prominence in the leadership literature is Eagly’s (1987) social role theory which proposes that external social pressures and cultural expectations guide individuals to adopt behaviors that are consistent with one’s gender. Individuals then internalize these expectations and become motivated to act consistently over time (see also Eagly and Karau, 2002). What constitutes “proper gender behavior” in a specific culture, then, could be interpreted as one of many variants of cultural information having been transmitted intergenerationally and having survived because it was adopted by the majority. Therefore, men and women engage in behaviors that ascribe to gender stereotypes largely because these distinct roles have been transmitted through the generations. Consider the case of empathic behaviors. Like all trait-based behaviors, these vary between individuals. Second, they compete against other behaviors such as selfishly looking after oneself at the detriment of others. And third, these behaviors are imitable and thus can be passed on to members of the next generation who benefitted directly from these empathic behaviors. As such, one would expect empathy to be transmitted culturally similar to how it is transmitted genetically. Under cultural evolution, one could surmise that women’s empathy advantage over men evolved through the transmission of social expectations and reinforced by the actual empathic behaviors of female caregivers toward group members. Empathy, as shown earlier, comprises a multitude of constructs some of which are behavioral (e.g., helping), affective (e.g., personal distress), and cognitive (e.g., perspective taking; Davis, 1980). Collectively, each of these can be seen as a variant of information about a person. Due to biased transmission, it is possible that some variants outshone others and became accepted as information worthy of being communicated to others. Regardless of how these cultural variants competed with one another, the broader core idea or cultural package of “women-are-more-empathic-than-men” was reconstructed time and time again. Research shows that in most societies, females are the more person-oriented sex (see pp. 92–125 in Pinker, 2008) and female leaders in particular are more communal (e.g., affection, interpersonal sensitivity) compared to their agentic male peers (e.g., assertiveness, dominance; Eagly, 1987). Although differences in women’s empathy advantage over men vary cross-culturally (e.g., Costa et al., 2001; Schmitt et al., 2008), the existence of such an advantage seems undeniable. If one accepts that women-as-empathizers constitutes one package, then *perceiving* them in this light might be another package that evolved through reinforcement. Should the case be that women continue to behave more empathically than men over time, then the collective expectation will most certainly perpetuate this notion.

Cultural information is not obtained exclusively from our direct experiences but also through vicarious learning. Researchers have contributed a wealth of knowledge regarding both the nature of stereotypes and the impact they have on decision making, while they have only begun to ask how cultural stereotypes form in the first place. In a recent experimental study, Martin et al. (2014) reconstructed the process through which social information is passed on repeatedly from one individual to another, i.e., via a linear diffusion chain. They first presented one participant (Generation 1) with images of various cartoon-like alien beings each having different shapes and colors, as well as different traits used to describe people (e.g., arrogant, curious, tidy). They then asked the participant to recall as much information about the aliens as possible and to relay these “alien-attribute packages” to a second participant (Generation 2), and so on, until communication was transmitted sequentially to a seventh participant (Generation 7). Therefore, the study’s social-transmission element involved taking the recalled information from one participant and using it as training material for the next generation. Findings showed that when social information is conveyed sequentially, people improved in their ability to remember the attributes associated with a social target because the task became increasingly simplified through both the loss of some attributes escaping memory and the development of a systematic categorical structure. By the time the information had reached the last generation, for example, blue aliens were predominantly “sensible,” whereas green ones were “vulgar,” even when direct experience with these was absent. As such, Martin et al. (2014) demonstrated that complex and random information about people becomes simplified, consistent, and systematic as it passes through communicators’ cognitive limitations and biases. Through a process of “cumulative cultural evolution” (p. 1778), this information develops into a form that could be easily retrieved and accurately transmitted. With women’s rising participation in upper managerial echelons, and with some women assuming positions that had never before been occupied by a member of their sex, there is a risk that new stereotypes may originate and evolve.

Thus far, we employed multiple evolutionary approaches to explain why women tend to be selected over men to lead in times of crisis and we have made a reasonable case for empathy’s role in this regard. As culture evolves, however, so do our genes and most would agree that genes and culture play a dual role in explaining behavior. Much value would therefore be gained if glass cliff phenomena were explained using both genetic and cultural evolutionary approaches in tandem, a case where the whole is greater than the sum of its individual parts. The latest evolutionary approach to have been developed, namely gene-culture coevolution, promises to offer such added value to our understanding, a prospect we turn to in greater detail next.

## Avenues for Future Research

Scholars in management and organizational psychology now urge researchers to go beyond transformational leadership and begin considering emotional traits and abilities, and biologically based arguments (Boyatzis, 2015). The spirit of this article addresses this call. Before rash conclusions are made to elect a leader on the sole basis of sex, however, we invite researchers to test our propositions. This could be done using best practices in experimental vignette methodology (Aguinis and Bradley, 2014) by varying the sex and empathy level of prospective leader candidates, as well as the crisis type and intensity. Researchers could then replicate laboratory findings with historical records from real companies or use members from board of directors as research participants to increase ecological validity. While we have explained how women’s perceived empathy could be the driving force for glass cliff appointments, we did not tackle the mechanism by which this is thought to occur. For example, the cascade of events surrounding glass cliffs could conceivably unfold as follows. First, an organizational crisis is experienced in type and intensity that sets off various aversive emotions among the firm’s stakeholders. Recognizing a need to change strategic direction, the firm’s board of directors establishes criteria for CEO selection and begins to search for the right fit, experience, and leadership traits and behaviors from a pool of potential candidates. Finally, the board selects those deemed to have the right mix of qualifications and proceeds with interviews and reference checks that culminate in the choice of a new CEO hire. Currently, such a decision-making process involved in selecting a leader following a crisis remains to be studied.

To interpret glass cliffs, researchers may also apply principles of gene-culture coevolution or what Boyd and Richerson (2005) refer to synonymously as “dual-inheritance theory.” Seen as a hybrid between cultural evolution and evolutionary psychology, gene-culture coevolution investigates the transmission of genes and cultural traits from one generation to the next by treating the two interdependently (Feldman and Cavalli-Sforza, 1981). It is the only evolutionary approach that explores the interaction of genes and culture. Like cultural evolutionists, gene-culture coevolutionists treat culture as a pool of socially learned and transmitted ideas and beliefs that are in constant flux. Like evolutionary psychologists, they agree that learning relies on biologically evolved knowledge-gaining structures (Laland and Brown, 2011). However, unlike behavioral ecologists, gene-culture coevolutionists believe that gene-culture interactions can lead to non-adaptive or even maladaptive outcomes. Finally, unlike evolutionary psychologists, they are more accepting of the idea that genetic evolution can be fast while cultural evolution can be slow (Bolhuis et al., 2011). Any treatment of glass cliffs from this latest approach would require scholars to track changes in allele frequencies resulting from changes in cultural practices. In other words, as men continue into the future to toil at work that was once reserved for women (e.g., childcare) and, as women continue to labor in traditional male occupations (e.g., organizational leadership), both sexes may 1 day be seen as equally empathic and therefore equally suited to handle crises of an interpersonal nature. While such an endeavor is challenging, we encourage future scholars to inspect glass cliff appointments through the lens of gene-culture coevolution since some predict that this evolutionary paradigm promises to become the most rewarding (Laland and Brown, 2011; Brown and Richerson, 2014).

Lastly, recent work has reported that glass cliffs may be generalizable to other minorities (Cook and Glass, 2014). Management researchers noted long ago that women and minorities face similar impediments in rising to the executive suite, and one of their explanations is that minorities, like women, see themselves as “tokens” whose performance is hindered by the pressure of visibility (Kanter, 1977; Morrison and von Glinow, 1990). One theory that could explain whether empathy lies at the heart of these appointments among minorities is the approach-inhibition theory of power (Keltner et al., 2003). This theory holds that power influences behavior by causing changes to occur between approach and inhibition systems. Approach systems are associated with reward seeking (e.g., achievement), whereas inhibition systems are associated with threat avoidance (e.g., heightened vigilance, anxiety). High-power individuals are more likely to activate approach systems because power enables them to seek reward with little concern that others might interfere. The lack of power emanating from a diminished social position—one that often characterizes minorities and women—may affect one’s empathy. Being more empathic may provide these marginalized individuals with certain advantages in the workplace compared to those who are less empathic, such as interpreting more accurately others’ feelings and intentions. Recent work has shown that individuals high in social power express less empathy than those low in power (Côté et al., 2011). As such, minorities might be perceived as being more empathic relative to non-minorities due to their power disadvantage and might become prone to being selected to lead when situations turn bleak. Knowledge of glass cliffs among minorities, or more specifically the perceptions that people harbor about minorities’ level of empathy relative to that of the majority, is limited and in need of development given the changing demographic landscape at work (Cook, 2013; Kulich et al., 2014).

## Implications for Theory and Praxis

We have articulated that people’s perception of women’s empathy affects their decisions to hire women leaders for risky positions. We examined this conundrum by setting aside some known and valid discriminatory causes for glass cliffs to reveal a more basic reason explaining why women leaders tend to be chosen to handle conflict, one rooted in a Darwinian heritage. In the first place, our work contributes to theoretical development. By discussing evolutionary and cultural processes in the same breath, we hope to have succeeded in portraying a more complete model of human interaction. This convergence of approaches also has reciprocal benefits, namely that social psychology models can complement biological mechanisms that support socialized behaviors (Decety and Lamm, 2006), with the end result being the generation of new hypotheses. Our work is also relevant for practitioners. First, an important reason why women are appointed to glass cliff positions remains unknown, and any anti-discriminatory legislation that is pursued will fall short of achieving its goal if the source of bias is not determined. Second, understanding implicit theories about women ought to reduce discriminatory biases against other minority groups that are in the same predicament (Fiske et al., 2002). Third, the glass cliff is believed to be partially responsible for reducing the tenure of women in executive positions due to burnout and stress (Bruckmüller et al., 2014). Leadership is itself a stressful endeavor so one can only imagine the stress incurred to lead an organization through a crisis. Therefore, a better understanding of the glass cliff could help protect women’s psychological and physical health. Fourth, glass cliffs not only place women in positions where the likelihood of failure is high, but also contribute to the proliferation of stereotypes that tarnish their true leadership ability. Research has shown that decision makers view both women and minorities as less capable leaders than typical white males (Rosette et al., 2008; Carton and Rosette, 2011). As such, learning more about what gives rise to the glass cliff could be essential in eliminating such stereotypes. Finally, by making people aware of the their decision-making biases, one can aspire to combat them and give qualified applicants equal opportunities for leadership regardless of sex.

### Conflict of Interest Statement

The authors declare that the research was conducted in the absence of any commercial or financial relationships that could be construed as a potential conflict of interest.
